# Age-related obesity and type 2 diabetes dysregulate neuronal associated genes and proteins in humans

**DOI:** 10.18632/oncotarget.4904

**Published:** 2015-08-18

**Authors:** Mehran Rahimi, Manlio Vinciguerra, Mojtaba Daghighi, Behiye Özcan, Vishtaseb Akbarkhanzadeh, Fareeba Sheedfar, Marzyeh Amini, Tommaso Mazza, Valerio Pazienza, Mahdi M. Motazacker, Morteza Mahmoudi, Felix W. M. De Rooij, Eric Sijbrands, Maikel P. Peppelenbosch, Farhad Rezaee

**Affiliations:** ^1^ Faculty of Medical Science, University Medical Center Groningen, University of Groningen, Groningen, The Netherlands; ^2^ Institute for Liver and Digestive Health, Division of Medicine, University College London (UCL), London, UK; ^3^ Gastroenterology Unit, IRCCS Casa Sollievo della Sofferenza, San Giovanni Rotondo, Italy; ^4^ Department of Biomedical Engineering, University Medical Center Groningen, University of Groningen, Groningen, The Netherlands; ^5^ Department of Endocrinology, Erasmus Medical Center, Rotterdam, The Netherlands; ^6^ Institute Center-45, Medical Center, University of Amsterdam, The Netherlands; ^7^ Department of Physiology, Radboud University Medical Center, Nijmegen, The Netherlands; ^8^ Department of Epidemiology, University Medical Center Groningen, University of Groningen, Groningen, The Netherlands; ^9^ Bioinformatics Unit, IRCCS Casa Sollievo della Sofferenza, San Giovanni Rotondo, Italy; ^10^ Department of Clinical Genetics, Academic Medical Center, Amsterdam, The Netherlands; ^11^ Department of Pediatrics, Stanford University School of Medicine, Stanford, CA, United States; ^12^ Department of Nanotechnology and Nanotechnology Research Center, Faculty of Pharmacy, Tehran University of Medical Sciences, Tehran, Iran; ^13^ Department of cardiovascular genetics, Metabolism, Erasmus Medical Center, Rotterdam, The Netherlands; ^14^ Department of Gastroenterology and Hepatology, Erasmus University Medical Center, University of Rotterdam, Rotterdam, The Netherlands; ^15^ Department of Cell Biology, University Medical Center Groningen, University of Groningen, Groningen, The Netherlands

**Keywords:** aging, obesity, diabetes, age-related diabetes neuropathy, pancreas

## Abstract

Despite numerous developed drugs based on glucose metabolism interventions for treatment of age-related diseases such as diabetes neuropathies (DNs), DNs are still increasing in patients with type 1 or type 2 diabetes (T1D, T2D). We aimed to identify novel candidates in adipose tissue (AT) and pancreas with T2D for targeting to develop new drugs for DNs therapy.

AT-T2D displayed 15 (e.g. *SYT4* up-regulated and *VGF* down-regulated) and pancreas-T2D showed 10 (e.g. *BAG3* up-regulated, *VAV3* and *APOA1* down-regulated) highly differentially expressed genes with neuronal functions as compared to control tissues. ELISA was blindly performed to measure proteins of 5 most differentially expressed genes in 41 human subjects. SYT4 protein was upregulated, VAV3 and APOA1 were down-regulated, and BAG3 remained unchanged in 1- Obese and 2- Obese-T2D without insulin, VGF protein was higher in these two groups as well as in group 3- Obese-T2D receiving insulin than 4-lean subjects. Interaction networks analysis of these 5 genes showed several metabolic pathways (e.g. lipid metabolism and insulin signaling).

Pancreas is a novel site for APOA1 synthesis. VGF is synthesized in AT and could be considered as good diagnostic, and even prognostic, marker for age-induced diseases obesity and T2D. This study provides new targets for rational drugs development for the therapy of age-related DNs.

## INTRODUCTION

The homeostasis of blood glucose levels is maintained by both insulin and glucagon synthesis, which is functionally tightly regulated by the pancreatic β-cells and α-cells respectively. When blood glucose levels rise (hyperglycemia), pancreatic β-cells synthesize and secrete insulin to the bloodstream to regulate glucose levels. On the contrary, when circulating glucose levels fall below normal values (hypoglycemia), pancreatic α -cells produce and release glucagon to the bloodstream to tune up glucose concentrations via conversion of stored glycogen into glucose and its release into the circulation [1, 2]. Thus, a disruption in blood glucose regulation leads to the hyperglycemia state so-called diabetes. There are two types of aged-related [3, 4] diabetes; type 1 diabetes (T1D) and type 2 diabetes (T2D). Diminished or dysregulation of insulin by β-cells in T1D and T2D respectively are caused by cell aging process. T1D is an autoimmune disease that pancreatic β-cells are targeted to be broken down by antibodies produced by immune B cells. In this regard, the main production site of insulin is diminished by β-cells [5–9]. In contrast, in age-related-T2D the pancreatic β-cells are still active and do synthesize insulin but at dysregulated levels and not sensitive enough [10–13]. In a normal state, there is a homeostatic balance and cross talk between skeletal muscle, liver and adipose tissue (AT) organs with respect to glucose levels, which is strictly under control of insulin [14]. In this regard, insulin 1- promotes glucose uptake and inhibits lipolysis by the skeletal muscle, 2- stimulates glycogenesis and suppress glucose formation and release from the liver, and 3- stimulates adipogenesis and prevent lipolysis in AT-associated adipocytes [14]. In an obese state, insulin shows a function completely opposite to the normal state in these three organs. Moreover, it has been recently shown that other cells such as adipocytes, macrophages also synthesize insulin [15]. Patients with either T1D or T2D diabetes are at high risk for serious complications such as cardiovascular diseases (CVDs) [16] and diabetic neuropathy (DN) [17–23]. Obesity plays an important role in the development of T2D and its complications via the promotion of an inflammatory state [16]. In this regard, Systems biology could be a solution to find etiology of aged-related diseases such as T1D, T2D, neurodegenerative and cardiovascular diseases [24].

DNs are the most common micro-vascular complications that occur in both T1 and T2 diabetes (40–60%) [18–20, 25, 26]. DNs-associated syndromes are classified into two groups; diffuse and focal. The former neuropathies are usually chronic and more common among diabetic patients, while focal neuropathies are acute [25]. In general, DNs occur in progressive damages of neuronal cells that commonly appear with symptoms such as severe pain, loss of sensation and disability [19, 22, 25].

Despite the general agreement that hyperglycemia plays a key role in initiation and development of DNs, the underlying mechanisms for the development of DNs is unknown and remains to be investigated [19, 22, 27]. Numerous studies have shown that hyperglycemia leads to the overload of electron transport chain in mitochondria and subsequent oxidative stress. The imbalance in the mitochondrial redox state, in favor of oxidation conditions, leads to the accumulation of reactive oxygen species (ROS). Subsequently, oxidative stress affects several metabolic pathways [such as insulin signaling pathway and protein kinase C (PKC) pathway; advanced glycation end products (AGEs) and mitogen activated protein kinase (MAPKs)] associated with glucose metabolism with enhanced oxidative stress and, in turn, inflammatory reactions [20, 21, 25, 28–32].

Although numerous drugs and therapies have been proposed to treat the DNs, the majority of these drugs and treatments were restricted to target the redox and glucose metabolic pathways [20, 32]. None of the drugs that targeted glucose metabolic pathways showed any efficient effect in the treatment of DNs [19, 22]. Also, despite identification of the genes [19] and metabolic pathways associated with DNs, the molecular causes of DNs events still occur in a large scale. These studies suggest that beside hyperglycemia other genetic components and factors may contribute to the development of DNs. For instance, tight links between oxidative stress and obesity were recently reported, which may provide new insight for investigation of the genes associated with DNs [21]. Meijer *et al.* have recently shown that metabolic dysfunction leads to the adipocytes hypertrophy (the main cause of obesity), which primes inflammation in AT [15, 16] and, in turn, is implicated in pathophysiological states such as energy storage disruption within adipocytes (e.g. triglycerides breakdown, FFAs, glycerol et cetera), insulin resistance (IR), and hyperglycemia as hallmark of T2D.

Feldman *et al*. reported several pathways involved in lipid, carbohydrate and energy metabolism modulating gene expression patterns in peripheral nerves on BKS db/db mouse sciatic nerve. Although this study support the hypothesis that hyperglycemia play an important role in nerve damage, lipid metabolism may involve neuronal damage and development of DNs as well [19]. Since both pancreas and AT are key tissues involved in age-induced T2D and its complications, this study was designed to investigate whether T2D alters the expression of genes with a function in neuronal processes in human pancreas and AT and that might be implicated in the insurgence of DNs.

## RESULTS

### The effect of type 2 diabetes on genes with a neuronal function

#### Pancreas

**Figure 1:**
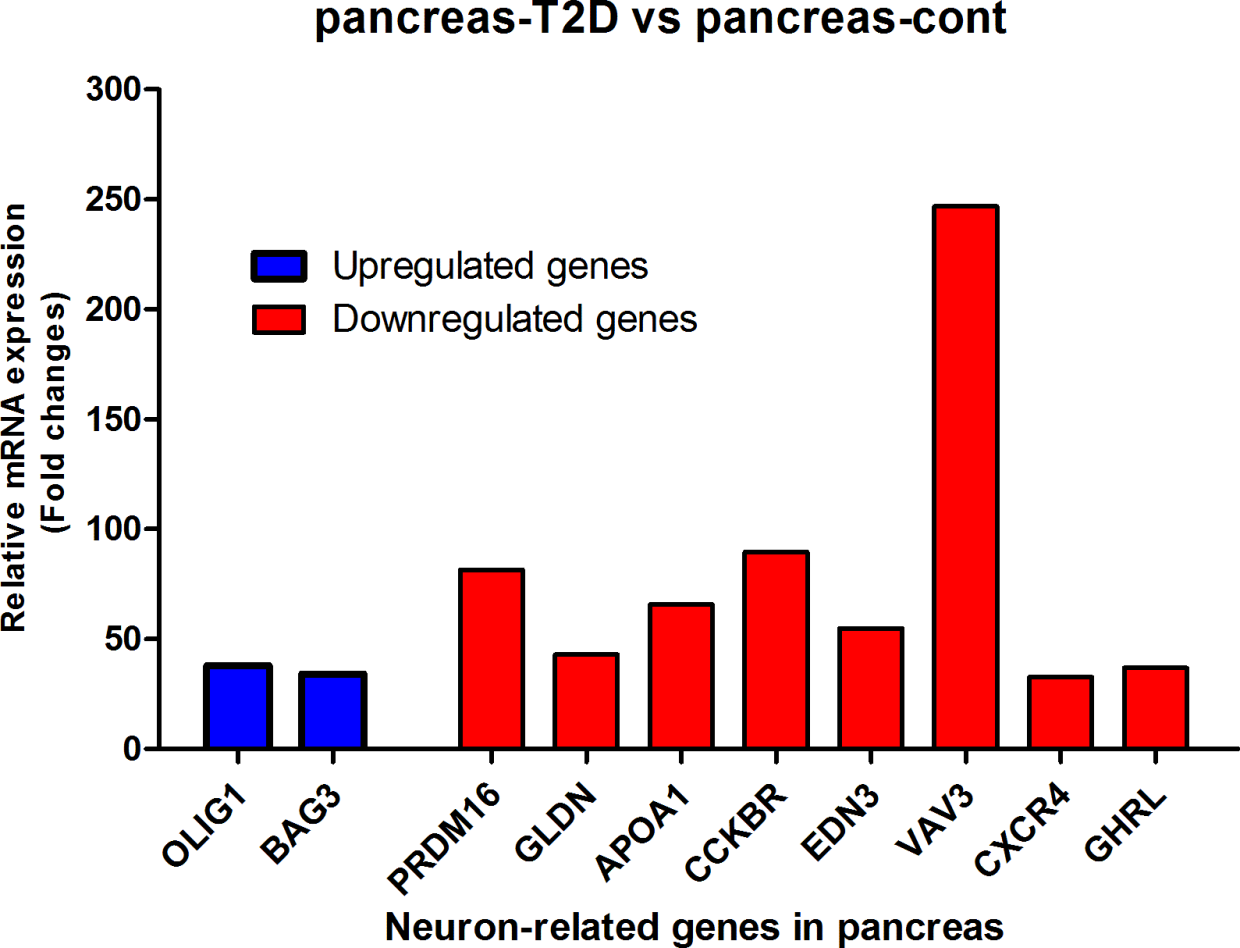
mRNA analysis of highly changed expression of 10 genes with a neuronal function in pancreas and pancreas with T2D After all corrections (see M & M), mRNA expression was expressed as ^2^log values to prevent false positive gene differences between two conditions. Subsequently, Fold-change between pancreas-T2D and pancreas-control was calculated and shown on the Y-axis and was obtained from three measurements.

**Figure 2:**
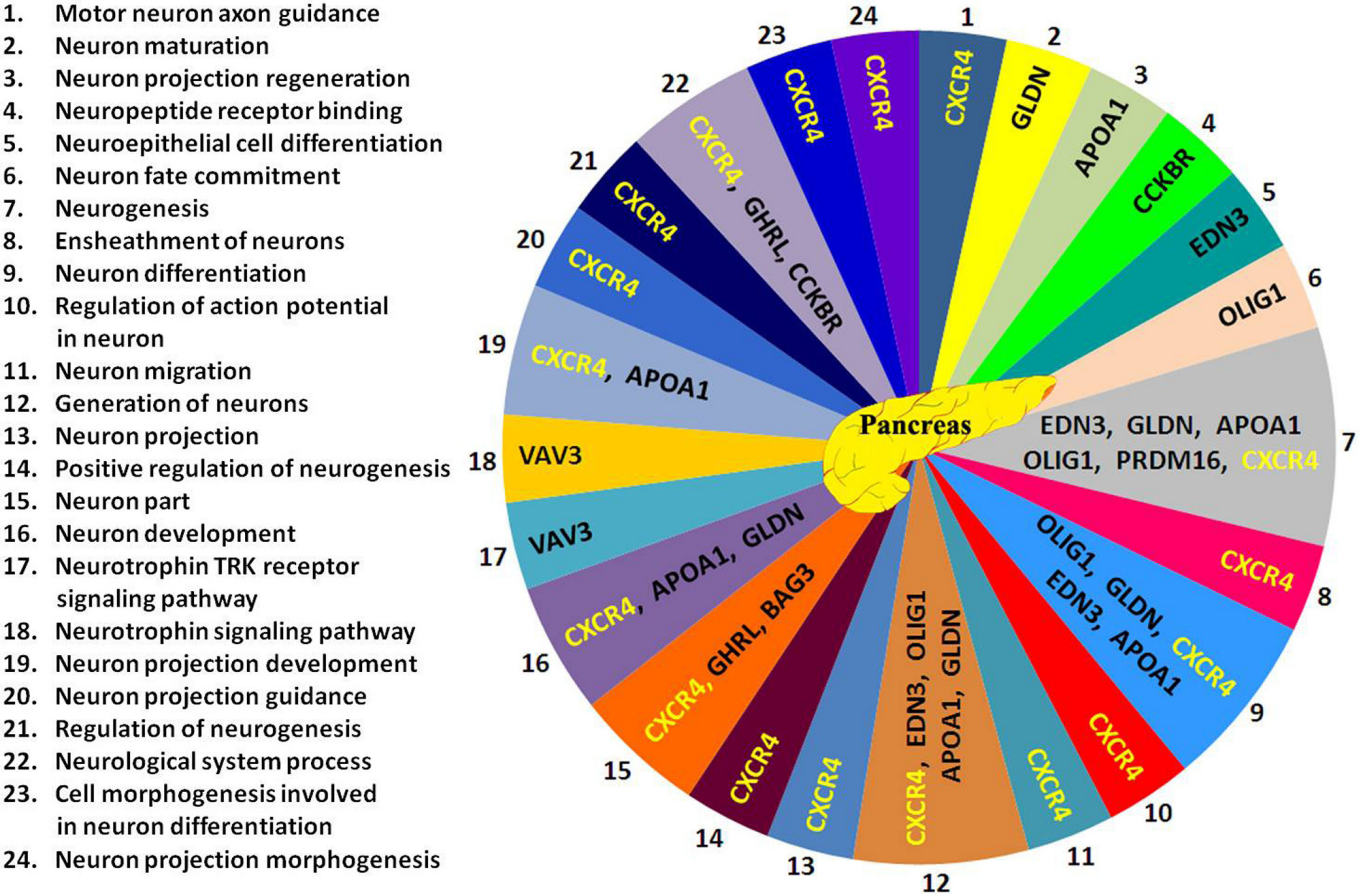
24 neuronal functions related to the 10 genes found in pancreas based on Gene Ontology database search Neuronal processes related to the 10 genes are numbered from 1 to 24 (left side of Figure) and corresponded with numbers 1 to 24 shown in pancreas Figure. Abbreviations are in Tables 1 and 2.

To investigate whether there are genes with a neuronal function that could show highly and clear changes in expression and that caused by age-induced T2D, we measured mRNA expression in human pancreas and adipose tissue (AT) with T2D. As depicted in Table 1 and Figure 1, two genes *OLIG1* and *BAG3* were highly up-regulated and eight genes *PRDM16*, *GLDNA*, *APOA1*, *CCKBR*, *EDN3*, *VAV3*, *CXCR4*, and *GHRL* were down regulated in pancreas with T2D as compared to pancreas control. Based on Gene Ontology database search, *OLIG1* gene shows functions in neuron fate commitment, neurogenesis, generation of neurons, and neuron differentiation as shown in Figure 2. Based on database search, *BAG3* gene showed only a neural function (i.e. neuron part). There were 24 neuron functions found for these 10 genes in pancreas of which chemokine C-X-C receptor *(CXCR4)* gene was shown to have a role in 17 neural functions based on Gene Ontology database (Figure 2). Among eight down-regulated genes in pancreas with T2D, *VAV3* gene was highest down-regulated gene with approximately 246-fold (Table 1 and Figure 1).

**Table 1:** The list of highly up-regulated and down-regulated genes with a neuronal function detected in human control pancreas and pancreas with type 2 diabetes (T2D) using mRNA expression

Protein ID	Protein description	pI	Mw (Da)	Gene name	Pan-T2D vs Pan-Cont (fold change)
Q8TAK6	Oligodendrocyte transcription factor 1	9.71	27,905	OLIG1	**38.0**
O95817	BAG family molecular chaperone regulator 3	6.46	61,595	BAG3	**34.2**
Q9UKW4	Guanine nucleotide exchange factor VAV3	6.64	97,776	VAV3	**−246.8**
P32239	Gastrin/cholecystokinin type B receptor	10.03	48,419	CCKBR	**−89.7**
Q9HAZ2	PR domain zinc finger protein 16	5.81	140,251	PRDM16	**−81,5**
P02647	Apolipoprotein A1	5.56	30,778	APOA1	**−65.7**
P14138	Endothelin-3	6.24	25,454	EDN3	**−55.0**
Q6ZMI3	Gliomedin	8.10	58,957	GLDN	**−43.0**
Q9UBU3	Appetite-regulating hormone	5.35	12,911	GHRL	**−37.0**
P61073	C-X-C chemokine receptor type 4	8.46	39,746	CXCR4	**−32.7**

Protein accession number, protein description, pI (isoelectric point), the Molecular weight (Mw), gene name of that protein, and fold change (Pancreas-T2D *vs* Pancreas-Control) are presented for each gene in this Table. The Mw expressed in Da. pI resulted from whole amino acid sequence of each protein and is derived from www.expasy.org.

#### Adipose tissue (AT)

**Figure 3:**
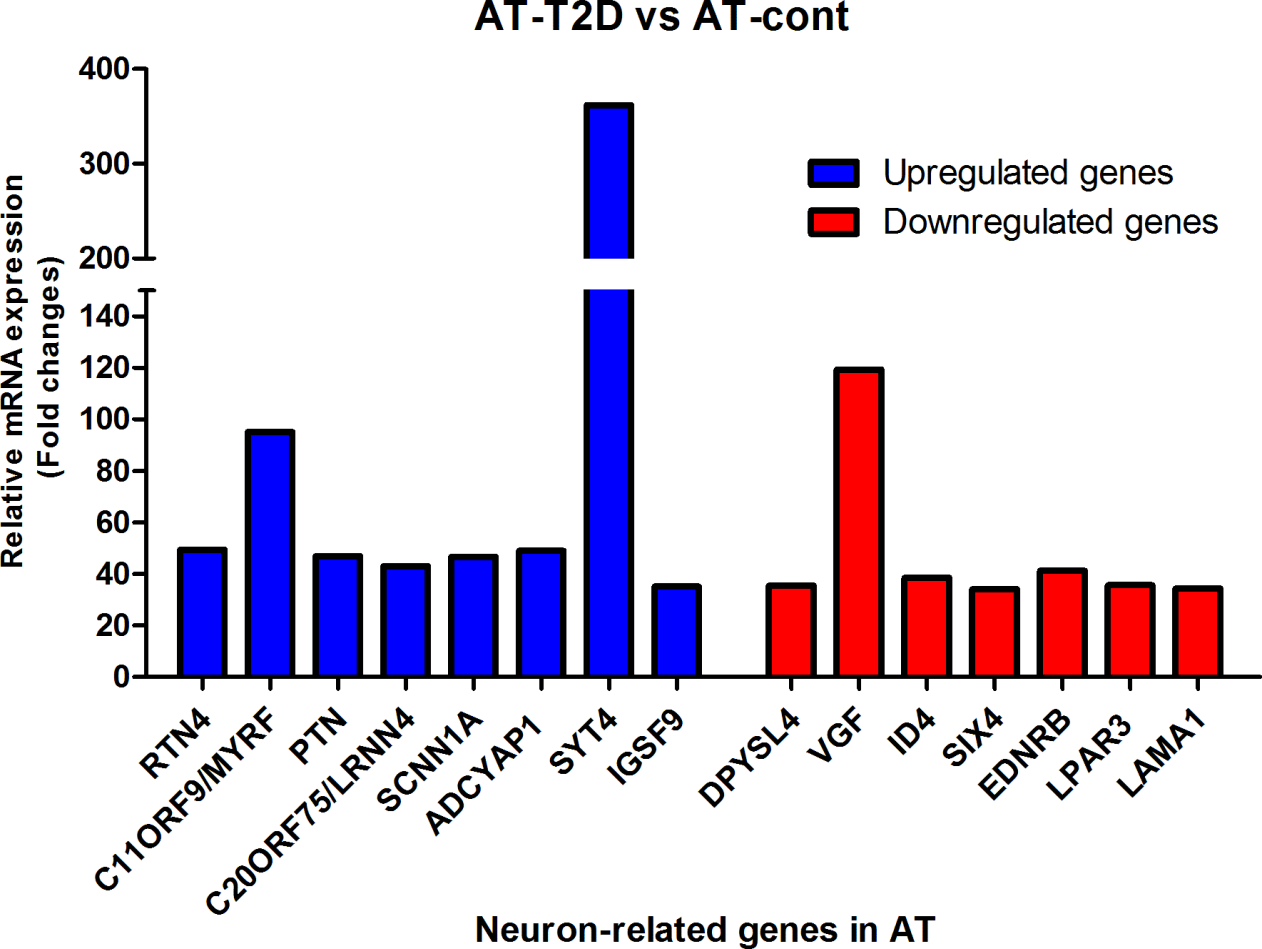
mRNA analysis of highly changed expression of 15 genes with a neuronal function in AT and AT with T2D After all corrections (see M & M), mRNA expression was expressed as ^2^log values to prevent false positive gene differences between two conditions. Subsequently, Fold-change between AT-T2D and AT-control was calculated and shown on the Y-axis and was obtained from two measurements.

**Figure 4:**
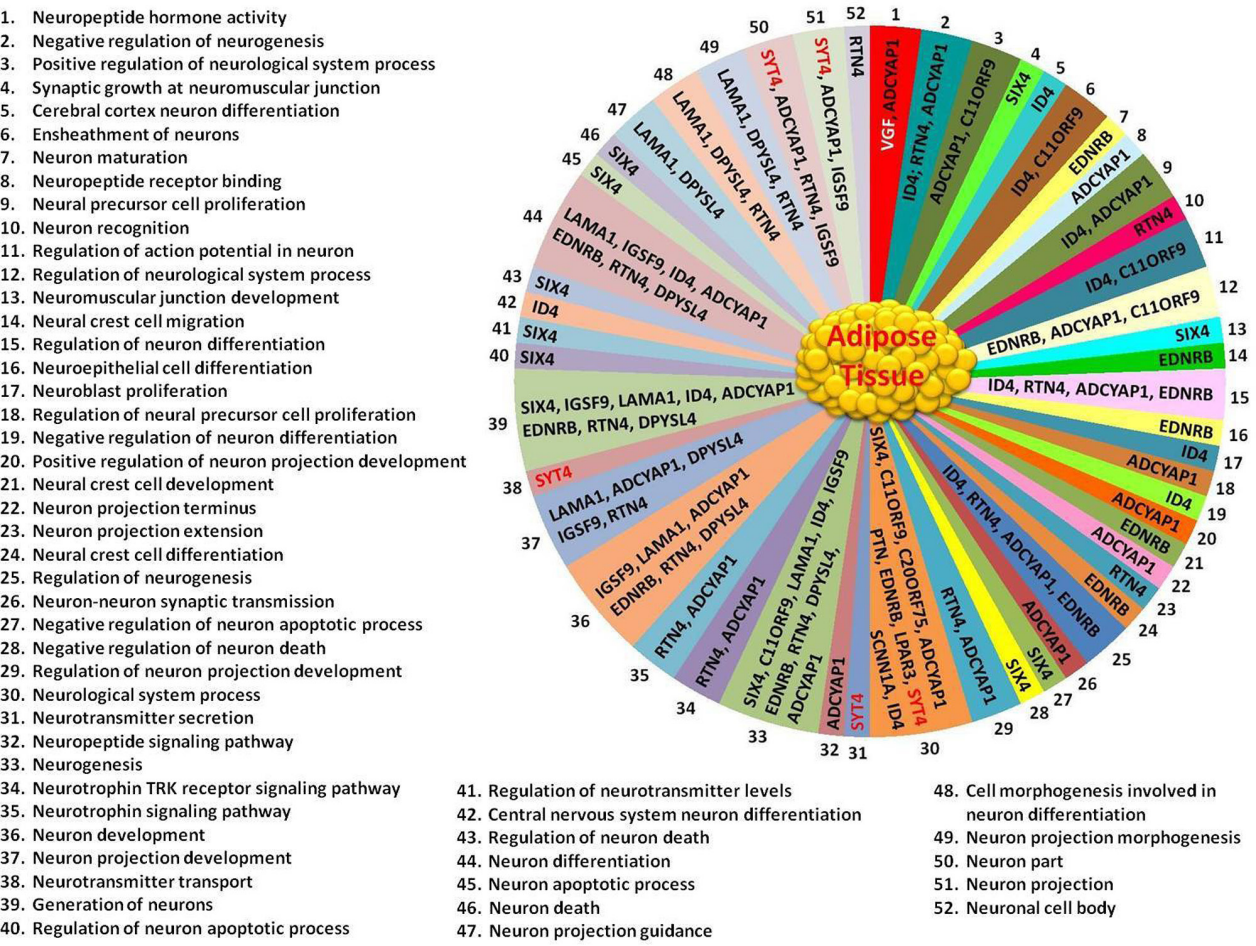
52 neuronal functions related to the 15 genes found in AT based on Gene Ontology database search Neuronal processes related to the 15 genes are numbered from 1 to 52 (left side and below of AT Figure) and corresponded with numbers 1 to 52 shown in AT Figure. Abbreviations are in Tables 1 and 2.

The expression of 15 genes were found to be highly altered in AT caused by age-induced T2D (Table 2 and Figure 3). From these 15 genes, 8 gens were highly up-regulated and 7 genes highly down-regulated (Table 2, and Figure 3). The expression of synaptotagmin-4 (*SYT4*) gene were tremendously high up-regulated (approximately 392-fold) in AT-T2D as compared to AT-control, while *VGF* (neurosecretory protein) is highly down-regulated (approximately 119-fold) Table 2 and Figure 3). Another gene, which is very highly up-regulated in AT-T2D as compared to AT-control was *MYRF* (Myelin regulatory factor) as displayed in Table 2 and Figure 3. The fold changes of all 15 genes were reported in Table 2. Based on Gene Ontology database search, 52 neural functions were found for these 15 genes (Figure 4). Although *VGF* gene was highly down-regulated, only one neural function (i.e. neuropeptide hormone activity) was shown to be linked with this gene (Figure 4). *SYT4* gene showed a link with 5 neural functions; 1- neurological system process, 2- neurotransmitter secretion, 3- neurotransmitter transport 4- neuron part and 5- neuron projection. Based on Gene Ontology search, *PTN* and *LRNN4* genes showed only link with one neural function (Figure 4) and both genes were up-regulated, while the other genes showed multiple neural functions.

**Table 2:** The list of highly up-regulated and down-regulated genes with a neuronal function detected in human control adipose tissue (AT) and AT-T2D using mRNA expression

Protein ID	Protein description	pI	Mw (Da)	Gene name	AT-T2D vs AT-CONT (fold change)
Q9H2B2	Synaptotagmin-4	8.72	47,958	SYT4	**362.08**
Q9Y2G1	Myelin regulatory factor	7.05	124,397	C11ORF9/MYRF	**95.06**
Q9NQC3	Reticulon-4	4.42	129,931	RTN4	**49.36**
P18509	Pituitary adenylate cyclase-activating	9.83	18,835	ADCYAP1	**48.92**
P21246	Pleiotrophin	9.66	18,942	PTN	**46.90**
P37088	Amiloride-sensitive sodium channel alpha	7.47	75,704	SCNN1A	**46.59**
Q8WUT4	Leucine-rich repeat neuronal protein 4	6.82	78,843	C20ORF75/LRNN4	**42.98**
Q9P2J2	Protein turtle homolog A	6.74	126,58	IGSF9	**35.06**
O15240	Neurosecretory protein	4.76	67,258	VGF	**−119.29**
P24530	Endothelin B receptor	9.15	49,644	EDNRB	**−41.21**
P47928	DNA-binding protein inhibitor ID-4	8.69	16,622	ID4	**−38.44**
Q9UBY5	Lysophosphatidic acid receptor 3	9.53	40,128	LPAR3	**−35.62**
O14531	Dihydropyrimidinase-related protein 4	6.64	61,878	DPYSL4	**−35.47**
Q7Z5W6	lamininA 1	7.02	60,711	LAMA1	**−34.41**
Q9UIU6	Homeobox protein SIX4	5.45	82,933	SIX4	**−34.09**

Protein accession number, protein description, pI (isoelectric point), the Molecular weight (Mw), gene name of that protein, and fold change (AT-T2D vs AT-Control) are presented for each gene in this Table. The Mw expressed in Da. pI resulted from whole amino acid sequence of each protein and is derived from www.expasy.org.

#### Pathway analysis

**Figure 5:**
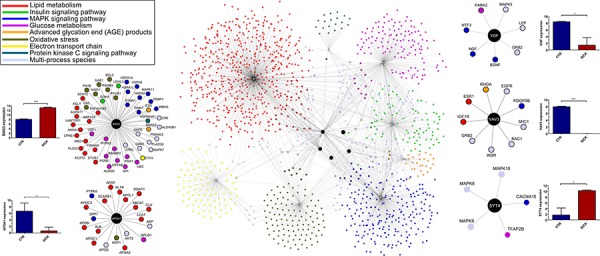
Functional interaction networks of the 5 most differentially expressed genes between AT and AT with T2D, and pancreas and pancreas with T2D Reviewed interactions of *SYT4*, *VGF*, *BAG3*, *APOA1*, and *VAV3* with genes participating to a list of critical biological processes and molecular functions (top-left) [source: BioGRID 3.2]. Nodes of networks are genes, while edges are interactions. Nodes are colored according to the functions/processes they participate in. Genes taking part in multiple processes are colored in cyan. Gene expression levels are represented by bars. Synaptotagmin-4 (*SYT4*), neurosecretory protein VGF (*VGF*), BAG family molecular chaperone regulator 3 (*BAG3*), apolipoprotein A-1 (*APOA1*), and guanine nucleotide exchange factor VAV3 (*VAV3*).

**Figure 6:**
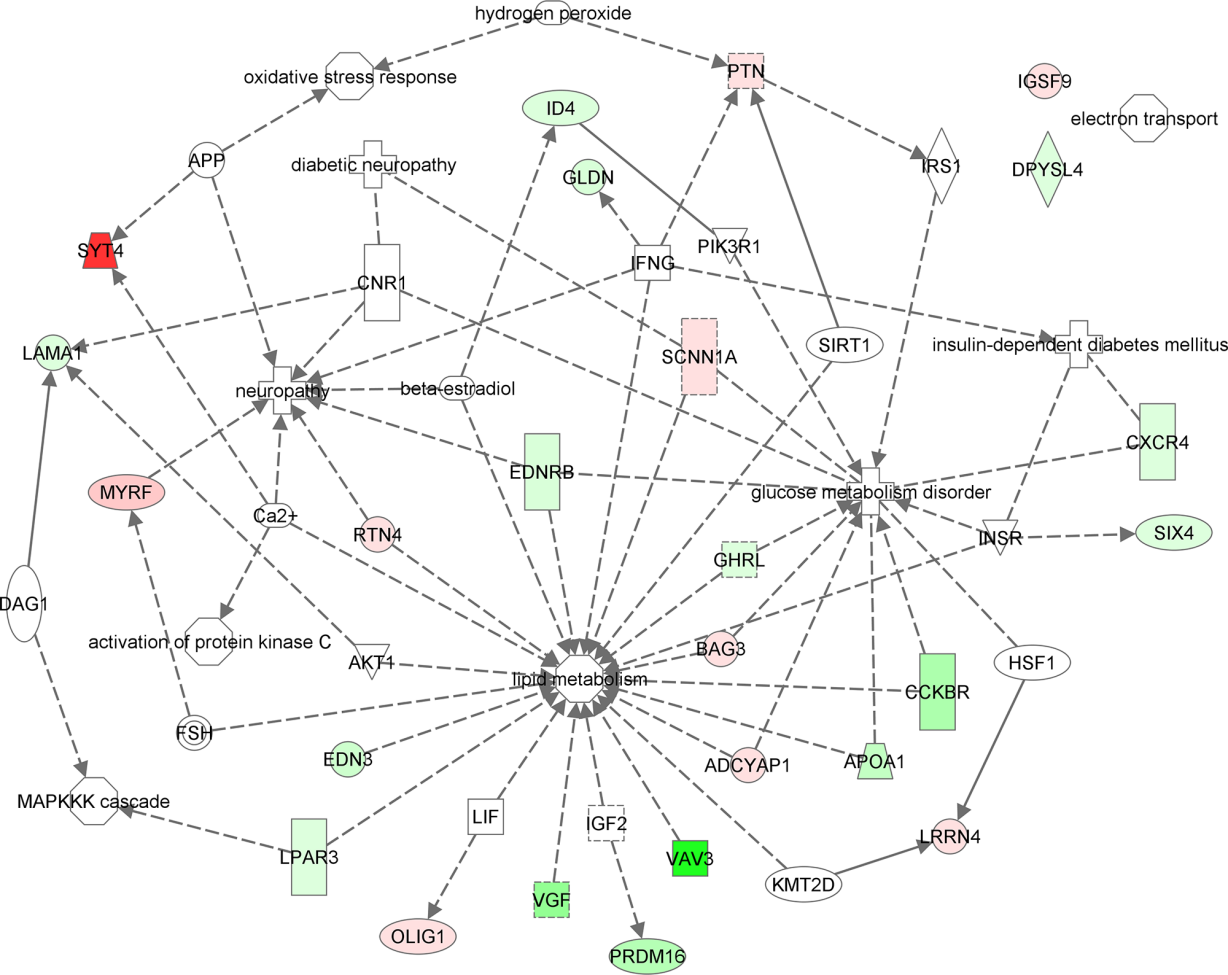
In-silico functional enrichment analysis of 25 differentially expressed genes between AT and AT with T2D, and pancreas and pancreas with T2D Functional network wiring 25 differentially expressed genes between AT and AT with T2D, and pancreas and pancreas with T2D drawn by Ingenuity Pathway Analysis. Colored glyphs are genes. Octagons and crosses are relevant functions and disorders, respectively. Edges represent interactions between genes or participation of genes to functions. Dashed edges are indirect interactions.

The *in-silico* functional analysis conducted on the list of 25 genes, led to the identification of several molecule-function/disease connections, as shown in Figure 5. Focusing on *SYT4*, *VGF*, *BAG3*, *APOA1*, and *VAV3* and on their tightly related interactors, which in turn participated to lipid metabolism, insulin, protein kinase C (PKC), advanced glycation end (AGE) products and MAPK signaling pathways, as well as to the electron transport chain, oxidative stress and glucose metabolism biological processes, we have drawn a global interaction network (Figure 6). It contained 198 (13.8%) multi-process genes, with 31 (*AKT2, CALM1, CALM2, CALM3, FOXA2, G6PC, HRAS, IKBKB, INSR, IRS1, MAPK1, MAPK3, MAPK8, MLYCD, PDK4, PRKAA1, PRKACA, PRKACB, PRKACG, PRKCG, PRKCZ, RAF1, SHC1, G6PD, PLA2G4A, SIRT1, SOD1, TNF, CASP3, EGFR* and *MAPK14*) genes participating to 3 processes, 7 (*FOXO1, INS, MAP2K1, MAPK9, PRKCA, PRKCB and NFKB1*) genes participating to 4 processes and 2 (*AKT1* and *PPARGC1A*) genes taking *EGFR, SHC1 and INSR* part in 5 processes. Due to its high node degree [33], *BAG3* resulted highly central in the whole graph. Among the others, it was connected to 8 multi-process genes, including *G6PD*. *APOA1* was mostly connected to lipid metabolism-related genes, with 3 multi-process genes, including *AKT2*. *SYT4* was connected to 5 genes of which only 3 were bi-process. Half of the *VAV3* interactors were multi-process, while *VGF* interacted with 7 genes, including 2 bi-process and *MAPK3*.

### The ELISA of SYT4, VGF, BAG3, ApoA1, and VAV3 proteins in plasma of 41 human subjects

To confirm the mRNA expression data derived from AT and pancreas with and without T2D, we measured five proteins SYT4, VGF, BAG3, ApoA1, and VAV3 in four different groups of 41 human subjects; 1- Lean (control), 2- Obese, 3- Obese with T2D without receiving insulin (obese+T2D-INS), and 4- Obese with T2D receiving insulin (obese+T2D+INS).

### SYT4 and VGF proteins

**Figure 7:**
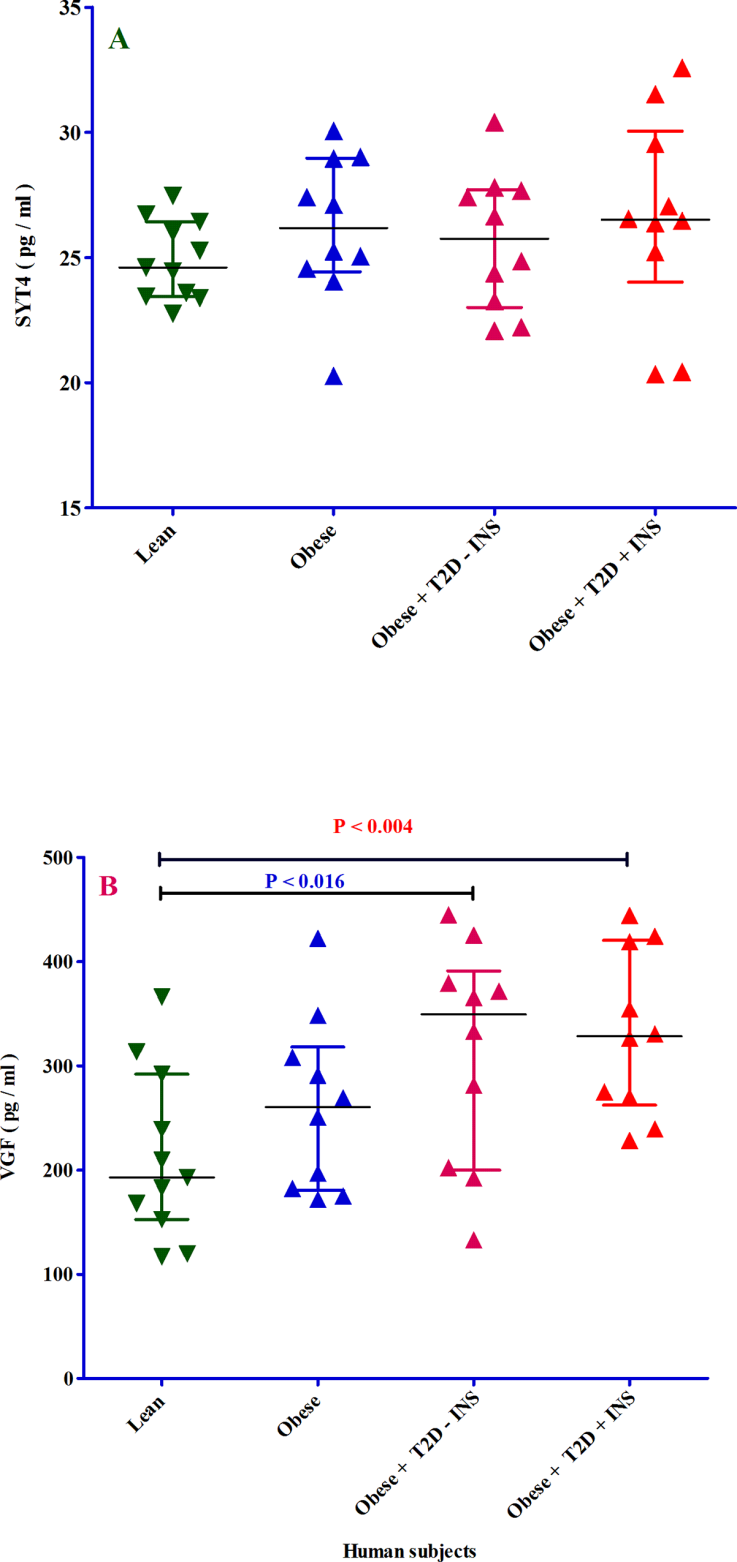
The analysis of SYT4 and VGF proteins in four different groups of 41 human subjects by ELISA Panel **A.** ELISA was used to measure synaptotagmin-4 (SYT4) protein in 4 different groups of patients; 1- lean (dark green triangle), obese (dark blue triangle), 3- obese with T2D without receiving insulin (light violet triangle) and 4- obese with T2D receiving insulin (red triangle). All samples were measured in duplicate and blind. The X-axis shows the four different groups of patients. The Y-axis shows the SYT4 concentration expressed as pg/ml. Panel **B.** ELISA was used to measure neurosecretory protein VGF (VGF) protein in 4 different groups of patients; 1- lean (dark green triangle), obese (dark blue triangle), 3- obese with T2D without receiving insulin (light violet triangle) and 4- obese with T2D receiving insulin (red triangle). All samples were measured in duplicate and blind. The X-axis shows the four different groups of patients. The Y-axis shows the VGF concentration expressed as pg/ml. The *p*-value resulted from one-way analysis of variance (ANOVA). *P* < 0.05 was accepted as statistically significant.

Although the protein concentration of SYT4 was not significantly changed in obese, obese-T2D with and without receiving insulin human subjects as compared to lean group, there was a clear up-regulation trend (Figure 7A), which is in agreement with SYT4 gene up-regulation. As depicted in Figure 7B, VGF protein concentration was significantly increased in both obese-T2D with and without receiving insulin subjects as compared to lean group. Although there was no significant difference between obese and lean group, the average of VGF protein concentration was clearly higher in obese subjects than lean group (Figure 7B).

### BAG3, APOA1, and VAV3 proteins

**Figure 8:**
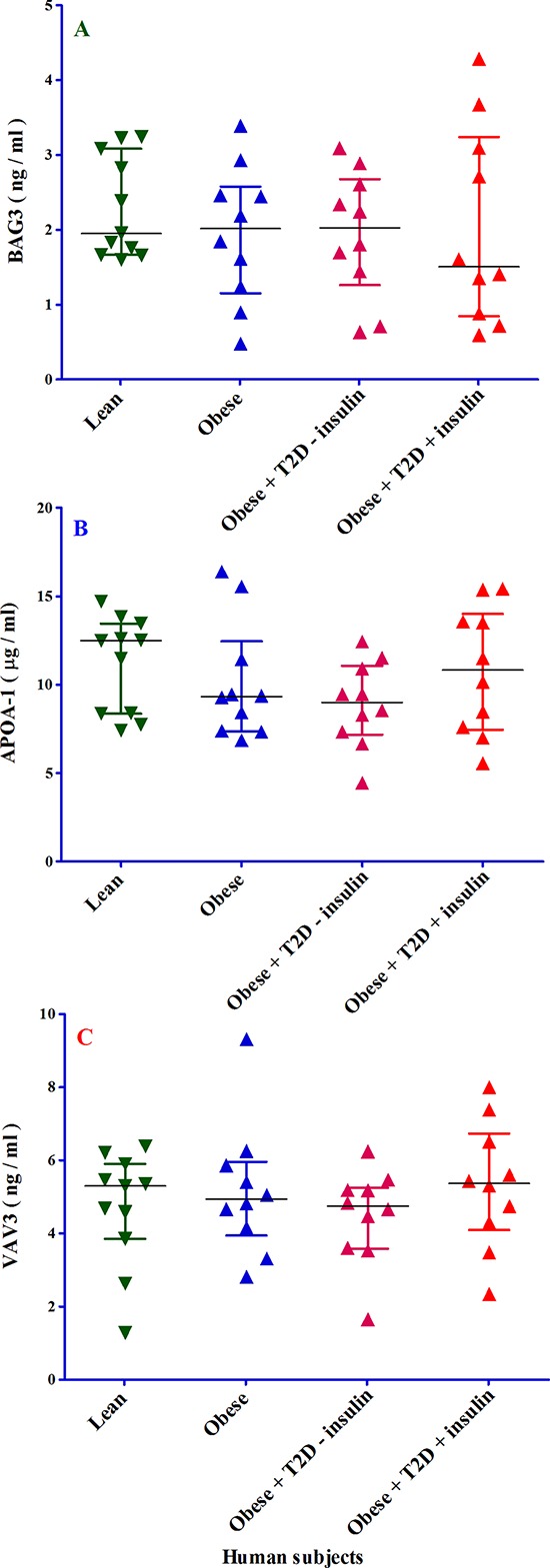
The analysis of BAG3, APOA1 and VAV3 proteins in four different groups of 41 human subjects by ELISA Panel **A.** ELISA was used to measure BAG family molecular chaperone regulator 3 (BAG3) protein in 4 different groups of patients; 1- lean (dark green triangle), obese (dark blue triangle), 3- obese with T2D without receiving insulin (light violet triangle) and 4- obese with T2D receiving insulin (red triangle). All samples were measured in duplicate and blind. The X-axis shows the four different groups of patients. The Y-axis shows the BAG3 concentration expressed as ng/ml. Panel **B.** ELISA was used to measure apolipoprotein A-1 (APOA1) protein in 4 different groups of patients; 1- lean (dark green triangle), obese (dark blue triangle), 3- obese with T2D without receiving insulin (light violet triangle) and 4- obese with T2D receiving insulin (red triangle). All samples were measured in duplicate and blind. The X-axis shows the four different groups of patients. The Y-axis shows the APOA1 concentration expressed as μg/ml. Panel **C.** ELISA was used to measure guanine nucleotide exchange factor VAV3 (VAV3) protein in 4 different groups of patients; 1- lean (dark green triangle), obese (dark blue triangle), 3- obese with T2D without receiving insulin (light violet triangle) and 4- obese with T2D receiving insulin (red triangle). All samples were measured in duplicate and blind. The X-axis shows the four different groups of patients. The Y-axis shows the VAV3 concentration expressed as ng/ml.

The plasma protein concentration of BAG3 was found to be similar in obese, obese-T2D without receiving insulin and lean subjects. However, there was a trend towards reduction in obese-T2D subjects receiving insulin as compared to the other three groups (Figure 8A). The protein levels of ApoA1 were not significantly reduced in all three experimental groups of patients as compared to the control lean group (Figure 8B). However, when obese-T2D subjects were injected with insulin, the plasma concentration of ApoA1 goes up comparing with obese and obese-T2D without injection of insulin as displayed in Figure 8B. Plasma VAV3 protein levels (Figure 8C) show a similar pattern as ApoA1, which is mirrored by down-regulation of *ApoA1* and *VAV3* genes. All statistical analysis obtained by ANOVA and ANCOVA was summarized in Table 3.

**Table 3:** Characteristics and protein concentrations of research population groups (*n* = 41)

*n* (%), mean (±SD)				
	Lean (BMI <25) (*n* = 11)	Obese (BMI >30) (*n* = 10)	Obese + T2D – INS (*n* = 10)	Obese + T2D + INS (*n* = 10)
Gender: *n* (Male %)	3 (27)	3 (30)	2 (20)	5 (50)
Age (years)	34.09 (±8.08)	45.20 (±6.23)	56.26 (±10.54)	67.18 (±6.97)
SYT4 (pg/ml)	24.95 (±1.64)	26.28 (±3.01)	25.71 (±2.66)	26.68 (±4.09)
VGF (pg/ml)	212.27 (±80.57)	261.20 (±85.74)	309.80 (±105.99)	330.60 (±78.93)
VAV3 (ng/ml)	4.75 (±1.64)	5.14 (±1.81)	4.53 (±1.31)	5.25 (±1.69)
apoA-1 (μg/ml)	11.20 (±2.72)	10.23 (±3.32)	8.88 (±2.35)	10.76 (±3.56)
BAG3 (ng/ml)	2.32 (±0.69)	2.01 (±0.90)	1.93 (±0.87)	2.06 (±1.29)

Subjects categorized into four groups including: lean (20.1 ≤ BMI ≤ 25.6 kg/m2), obese (BMI ≥ 30 kg/m2), obese with T2D without receiving insulin (obese+T2D-INS) and obese subjects with T2D receiving insulin (obese+T2D+INS). Protein concentrations were normally distributed and were presented as mean±SD.

## DISCUSSION

Aging can be expressed simply as “functionality reduction of biological process” or as “loss or reduction of the regulated cell function”. Of note, the aging process can be distinguished between early or premature aging and regulated (normal) or healthy aging. Thus, all diseases such as obesity, IR, diabetes, degenerative disease, and cancer could be just referred to aging or aging-related diseases. The discovery of the factors responsible for premature aging or healthy aging will provide us information to correct premature aging or extend healthy aging [23].

Although the underlying mechanisms for the development of human T1D (autoimmune disease) are completely different from T2D, insulin signaling pathway, glucose levels, and energy storage components are involved in both types of diabetes. Both age-induced T1D and T2D are associated with many complications such as DNs. We hypothesized that AT and pancreas could be involved in the development of DNs in age-related T2D. AT is important during the insurgence of insulin resistance in the frame of obesity and produce factors involved in energy metabolism, such as chemokines/cytokines, and the pancreas is implicated in insulin production. There is little known about the genes involved in DNs in these two organs (with or without T2D). This study was designed to select only genes with very highly up-regulated or down-regulated in AT and pancreas with age-related T2D (the cut off was adjusted to ≥ 32 (=2^5^) times). This selection criterion was chosen to prevent false positive gene differences between control and T2D in these two organs of human.

Overall, 10 genes with a neural function were found in pancreas-T2D of which 8 genes were highly down-regulated and 2 genes highly up-regulated. Although it is difficult from our studies to infer about the mechanism behind each gene in the development of DNs, we assume that these 10 genes could be potentially major players in DNs.

One of the functions of *CXCR4* is known to regulate neuronal signaling and promotes hippocampal-neuron survival [34–38], while the mutation affected the gene and, in turn, suppression of *CXCR4* function. Thus, down-regulation of *CXCR4* function may lead to the promotion of neuronal apoptosis. Of note, the *CXCR4* was down-regulated in pancreas-T2D. Neuronal survival and regulation of neuronal signaling by CXCR4 might be reduced in T2D patients, and this may play a critical role in the development of DNs. Moreover, Based on Gene Ontology database search, 17 hits with neuronal function were found for *CXCR4*.

To date, apolipoprotein A-1 (APOA1) was shown to be synthesized in the liver and in the small intestine and it is a major constituent protein of plasma high-density lipoprotein (HDL). APOA1 plays a major role in reverse cholesterol transport, as it has a high capacity to drive cholesterol efflux [39–41]. Intriguingly, *APOA1* was highly expressed in control pancreas but highly down-regulated in pancreas-T2D. Several studies have shown that the mutation in APOA1 is involved in the pathogenesis of polyneuropathy [42–46]. Also, APOA1 convincingly plays a role not only in the early stage but also in late neuropathy [42–46]. These studies support our finding that *APOA1* is highly down-regulated in pancreas-T2D, and it is involved in 6 neural processes.

*GHRL* is also known as appetite-regulating hormone; it is highly down-regulated in pancreas-T2D and shows 2 neural functions. GHRL has been reported to protect neurons from damage and to improve neurons survival [47]. This may suggest that a down-regulation of *GHRL* could result in a decreased neural protection and neural survival [47].

*OLIG1*, also known as oligodendrocyte transcription factor 1, is very highly up-regulated in pancreas-T2D with four neural function hits and is involved in the formation of oligodendrocytes and together with *OLIG2* establish precursor motorneuron (pMN) of embryonic neural tube [48]. Based on literature search, we could only find one study related to up-regulation of *OLIG1* and disease [49]. In this study, *OLIG1* was highly expressed in oligodendrogliomas and considered as biomarker for glial brain tumors. Thus, the up-regulation of *OLIG1* in pancreas-T2D suggests that new studies are crucial to better elucidate *OLIG1* function (s).

The major action of *VAV3* (also guanine nucleotide exchange factor (*GEF*)) is in angiogenesis and it has been recently shown that loss of *VAV3* in mice on chow diet resulted in liver steatosis and age-induced T2D [50]. Surprisingly, when the same mice were fed a high fat diet, they showed resistance against diet-induced obesity and metabolic syndrome [51]. However, our findings in humans do not support the data collected from mice deficient in *VAV3*. *VAV3* showed a down-regulated of approximately 246-fold in human pancreas with T2D as compared to control pancreas. This finding indeed suggests that the action of *VAV3* in human is completely different from that in mice with manipulated *VAV3* gene: the restoration of *VAV3* in pancreas-T2D could contribute to less severe DNs.

In AT-T2D, 8 genes were highly up-regulated and 7 genes down-regulated with neuronal hits. It is beyond the scope of this study to unravel and discuss the individual mechanism of each gene in the development of DNs. We assume that these 15 genes could be potentially major players in DNs. This study provides preliminary evidence for further investigations.

*SYT4* (Synaptotagmin-4) and *VGF* (neurosecretory protein) genes are dramatically up-regulated and down-regulated in AT-T2D, respectively. *SYT4* is mainly detectable in brain and neuroendocrine system and it has been suggested to have a neuroendocrine role [52, 53]. These authors showed that up-regulation of *SYT4* blocked the release of oxytocin, which in turn resulted in an obese phenotype [52, 53]. On the contrary, down-regulation of *SYT4* normalized oxytocin release, indicating that obesity and its afflictions are under regulation of hypothalamic neuropeptides [52, 53]. Based on our novel finding (overexpression of SYT4 in AT-T2D) and intriguing finding by Zhang *et al*. [52], we hypothesize that SYT4 can be used as a new biomarker for obesity and T2D and may be considered as prognostic marker for age-induced T2D, and negative regulation of *SYT4* could potentially repair or reduce the degree of DNs.

The main function of *VGF* (also called neuroendocrine regulatory peptide (*NERP*)) is synaptogenesis and they are mainly present in neuron and neuroendocrine cells [53–55]. Body fluid homeostasis was also maintained by VGF through regulation of vasopressin release [54, 55]. Moreover, VGF or VGF-derived peptides increase lipolysis in AT and in turn energy consumption in *VGF* deficient mice (knock-out), which are lean [56]. However, *VGF* was highly down-regulated in AT-T2D, which does not support the results obtained from *VGF* knock-out mice [56]. These contradictory results could be due to specific differences between mice and humans. Based on this evidence, we speculate that the *VGF* up-regulation (e.g. by an agonist) together with down-regulation of *SYT4* could restore the homeostatic balance between energy consumption and energy storage regulation, which could repair or reduce DNs.

Of note, the gene-expression data do not have enough power without the confirmation of gene product (i.e. protein). For this reason, five from 25 genes was selected to measure their protein levels. SYT4 and VGF proteins were chosen from AT-associated 15 genes, because *SYT4* gene was highest and *VGF* lowest expressed in AT-T2D respectively. Although plasma VGF protein concentration in three groups of patients did not correspond with *VGF* gene expression, both plasma SYT4 and VGF protein concentrations were increased and decreased in obese-T2D with insulin injection as compared to without insulin injection in human subjects respectively. The effect of insulin was also observed for BAG3, APOA1 and VAV3 in obese-T2D with and without insulin injection in human subjects. Based on these data, it is then logic to assume that insulin has an effect on the concentrations of these five proteins and this role of insulin could be considered as so-called “corrector’’. Importantly, *VGF* gene expression did not correspond to VGF protein levels and one reason could be that post translational modifications (PTMs) determine VGF protein levels and not transcriptional phase. Although we did not observe any significant differences between three patient groups and lean subjects with respect to SYT4, BAG3, APOA1, and VAV3, except for VGF protein, there was a trend between the expression of these four genes and their protein levels.

The majority of investigations and drug developments for DNs conducted so far focused on glucose metabolic pathways. Neuropathies still remains a great problem in patients with diabetes. The expression of *APOA1* in control pancreas and severe down-regulation in pancreas with T2D is very novel and important finding, which open new avenues on lipid metabolism, HDL, cardiovascular events, diabetes and its complications such as neuropathies, and will help to design new future studies. *GHRL* and *VAV3* are also expressed in pancreas and subjected to extreme level changes in pancreas-T2D. *SYT4* and *VGF* were synthesized in AT and the levels alter in AT-T2D dramatically. Based on this study, we conclude that not only diabetes but also its complications such as DNs are multifactorial diseases. Also, this study provides us a list of most important genes affected by diabetes involved in neuronal processes, which opens new avenues for new investigations for drug development (using agonists or antagonists) and new pathway(s) for the treatment of DNs.

## MATERIALS AND METHODS

### Human biopsies and gene expression procedures

Total RNA was isolated from human adult normal visceral AT and AT-T2D. Total RNA was collected from 5 pooled human normal pancreases and one pancreas with T2D provided by AMS Biotechnology (Amsbio, England). The quality and concentrations of RNAs were assessed in a Bioanalyzer (Experion), (Bio-Rad, USA/The Netherlands) using the Agilent RNA 6000 Nano kit (Agilent, The Netherlands). Amplification Kit was applied to amplify and label the RNA (Applied Biosystems, The Netherlands). Total RNA was reverse-transcribed into cDNA, and the concentrations were determined in NanoDrop. RNA Amplification Kit (Ambion, USA) was used to biotinylate cRNA according to the manufacturer's instructions. Samples were purified using the RNeasy kit (Qiagen, The Netherlands). Hybridization to the Sentrix Human Expression BeadChip, washing, and scanning were performed according to the BeadStation 500 manual (revision C). One BeadChip with 12 samples was used. Each slide was scanned immediately. After the scanning, the following steps were performed: 1: quality check; 2: background correction; 3: normalization to housekeeping genes; and 4: utile normalization of the data using Beadstudio Expression module v 3.2.7. After these 4 steps, a relative mRNA intensity of ^2^log was considered cut-off. Each pancreas sample was three times and AT samples two times measured. The methods were carried out in “accordance” with the approved guidelines.

### Human subjects and plasma collection

41 human subjects divided into four groups of 10 (except lean group 11 subjects), independent of age and gender, as follows: Group 1-Lean subjects with a body mass index (BMI) between 20.1 and 25.6 kg/m2, Group 2- Obese subjects with a BMI greater than or equal to 30 kg/m2, Group 3- Obese subjects with T2D without receiving insulin (obese+T2D-INS) and a BMI greater than or equal to 30 kg/m2 30 and Group 4- Obese subjects with T2D, receiving insulin (obese+T2D+INS) and BMI greater than or equal to 30 kg/m2. All patients were well-selected based on their BMI. Blood was drawn from each subject after informed consent by venipuncture into tubes containing Ethylene Diamine Tetra Acetic Acid (*EDTA*) as anticoagulant. Subsequently, plasma was isolated by centrifugation at 1500× g for 15 min at 4°C and stored at −80°C for further analysis.

### The measurement of protein concentrations of SYT4, VGF, BAG3, APOA1, and VAV3 by ELISA

96-well microtitration plates (NuncSorb) were coated with primary antibodies (100 μl at 5 μg/ml in PBS, 4°C). After four washes with PBS, the wells are saturated with BSA (200 μl, 3% in PBS, at least 2 h at RT). After four washes with PBS, 25 μl of each human serum sample were deposited in the wells and protein were allowed to bind to their specific mAb during 3 h at RT. All proteins not specifically bound were eliminated by four washes with PBS. Then the HRP-conjugated secondary antibodies were added (100 μl at 1 μg/ml in PBS, 45 min at RT). After four washes with PBS, 100 μl of HRP substrate (TMB: 3,3′,5,5′-tetramethylbenzidine; Thermo Scientific Pierce) were deposited and reaction was allowed during 15 min in dark (upon oxidation, TMB forms a water-soluble blue reaction product that can be measured spectrophotometrically at 650 nm). Reaction was then stopped by addition of 100 μl H2SO4 2 M (upon acidification, the reaction product becomes yellow with an absorbance peak at 450 nm). Absorbance at 450 nm (yellow) was monitored on a plate-reader device. Primary antibodies were purchased from SIGMA (SYT4, APOA1, BAG3), from Santa Cruz Biotechnology (VAV3) and Abcam (VGF). HRP-coupled secondary antibodies (anti-mouse, anti-goat, anti-rabbit) were purchased from Abcam.

### Interaction network-based pathway analysis

Genes belonging to *lipid metabolism*, *insulin*, *protein kinase C*, *advanced glycation end (AGE) products* and *MAPK* signaling pathways, together with those participating to the *electron transport chain*, *oxidative stress* and *glucose metabolism* biological processes were collected [57–58]. Considering that correlation of gene expression and protein to protein interactions have been shown to cluster genes of similar function [59], we queried UniProtKB, Mentha [60], STRING [61] and BioGRID [62] in search of any evidence of connection between these genes and *BAG3*, *APOA1*, *VGF*, *VAV3 and SYT4*. Interactions were represented as edges of graphs, while nodes denoted genes, as already explained in [63]. Genes were colored according to the biological processes/pathways they were participating in. Light blue nodes represented genes that took part to multiple processes and pathways. Differential expressions of *BAG3*, *APOA1*, *VGF*, *VAV3* and *SYT4* genes between normal and patient samples were evaluated by unpaired *student t-test* at significance level of α = 5%. A global view of the functional involvement of the 25 genes into the abovementioned critical processes and pathways has been further obtained by the Ingenuity Pathway Analysis workbench. A gene-function mixed network was generated, where edges corresponded to literature-confirmed scientific evidences of interaction between any two genes. Dashed edges represented indirect interactions. Green colored genes were down-regulated in our case-study, while red genes were up-regulated. Molecule shapes correspond to as many kinds of molecules, as for functions or diseases (octagons and crosses).

### Statistics

ANOVA was performed as statistical analysis to identify the protein levels of SYT4, VGF, VAV3, APOA1 and BAG3 for each BMI group (1- lean, 2- obese, 3- obese+T2D-INS and 4- obese+T2D+INS).

Analysis of covariance (ANCOVA) was used to identify differentially proteins concentrations of SYT4, VGF, VAV3, apoA1, and BAG3 across the four groups as controlling for the effects of confounding variables including age and gender. ANCOVA analysis was done using change of proteins concentrations in groups as dependent variable; ordinal variable of four groups (lean, obese, obese- T2D-INS, obese-T2D+INS) as a fixed effect and the effects of confounders were controlled in analysis as covariates (Table 3). The choice for a post-hoc analysis was due to having five proteins profiles across four groups. All statistical tests were two-sided and performed at a significance level of α = 5%. Data analyses were performed using SPSS statistical software (version 22; SPSS Inc., Chicago, USA).
